# Cannabidiol (Epidyolex®) for severe behavioral manifestations in patients with tuberous sclerosis complex, mucopolysaccharidosis type III and fragile X syndrome: protocol for a series of randomized, placebo-controlled N*-*of*-*1 trials

**DOI:** 10.1186/s12888-023-05422-3

**Published:** 2024-01-04

**Authors:** A. R. Müller, B. den Hollander, P. M. van de Ven, K. C. B. Roes, L. Geertjens, H. Bruining, C. D. M. van Karnebeek, F. E. Jansen, M. C. Y. de Wit, L. W. ten Hoopen, A. B. Rietman, B. Dierckx, F. A. Wijburg, E. Boot, M. M. G. Brands, A. M. van Eeghen

**Affiliations:** 1 Department of Pediatrics, Emma Children’s Hospital, Amsterdam Gastroenterology Endocrinology Metabolism, Amsterdam UMC Location University of Amsterdam, Amsterdam, The Netherlands; 2 ’s Heeren Loo Care Group, Amersfoort, The Netherlands; 3 Emma Center for Personalized Medicine, Amsterdam UMC, Amsterdam, the Netherlands; 4 United for Metabolic Diseases, Amsterdam, The Netherlands; 5 Department of Data Science and Biostatistics, University Medical Center Utrecht, Utrecht, The Netherlands; 6 Department of Health Evidence, Biostatistics, Radboud University Medical Center, Nijmegen, The Netherlands; 7 Child and Adolescent Psychiatry and Psychosocial Care, Amsterdam UMC Location Vrije Universiteit, Amsterdam, The Netherlands; 8 Amsterdam UMC, Amsterdam Neuroscience, Amsterdam Reproduction and Development, N=You Neurodevelopmental Precision Center, Amsterdam, The Netherlands; 9 Levvel, Center for Child and Adolescent Psychiatry, Amsterdam, The Netherlands; 10 Department of Human Genetics, Amsterdam UMC, Amsterdam, The Netherlands; 11 Department of Pediatric Neurology, Brain, University Medical Center Utrecht, Utrecht, The Netherlands; 12 ENCORE Expertise Center for Neurodevelopmental Disorders, Erasmus University Medical Center, Rotterdam, The Netherlands; 13 Department of Neurology, Erasmus University Medical Center, Rotterdam, The Netherlands; 14 Department of Child and Adolescent Psychiatry/Psychology, Sophia Children’s Hospital, Erasmus University Medical Center, Rotterdam, The Netherlands; 15 The Dalglish Family 22Q Clinic, Toronto, ON Canada; 16 Department of Psychiatry & Neuropsychology, Maastricht University Medical Center, Maastricht, the Netherlands

**Keywords:** N*-of-*1, Cannabidiol, CBD, Tuberous sclerosis complex, Sanfilippo disease, Mucopolysaccharidosis, Fragile X syndrome, Behavior, Intellectual disability

## Abstract

**Background:**

Many rare genetic neurodevelopmental disorders (RGNDs) are characterized by intellectual disability (ID), severe cognitive and behavioral impairments, potentially diagnosed as a comorbid autism spectrum disorder or attention-deficit hyperactivity disorder. Quality of life is often impaired due to irritability, aggression and self-injurious behavior, generally refractory to standard therapies. There are indications from previous (case) studies and patient reporting that cannabidiol (CBD) may be an effective treatment for severe behavioral manifestations in RGNDs. However, clear evidence is lacking and interventional research is challenging due to the rarity as well as the heterogeneity within and between disease groups and interindividual differences in treatment response. Our objective is to examine the effectiveness of CBD on severe behavioral manifestations in three RGNDs, including Tuberous Sclerosis Complex (TSC), mucopolysaccharidosis type III (MPS III), and Fragile X syndrome (FXS), using an innovative trial design.

**Methods:**

We aim to conduct placebo-controlled, double-blind, block-randomized, multiple crossover N*-of-*1 studies with oral CBD (twice daily) in 30 patients (aged ≥ 6 years) with confirmed TSC, MPS III or FXS and severe behavioral manifestations. The treatment is oral CBD up to a maximum of 25 mg/kg/day, twice daily. The primary outcome measure is the subscale irritability of the Aberrant Behavior Checklist. Secondary outcome measures include (personalized) patient-reported outcome measures with regard to behavioral and psychiatric outcomes, disease-specific outcome measures, parental stress, seizure frequency, and adverse effects of CBD. Questionnaires will be completed and study medication will be taken at the participants’ natural setting. Individual treatment effects will be determined based on summary statistics. A mixed model analysis will be applied for analyzing the effectiveness of the intervention per disorder and across disorders combining data from the individual N*-of-*1 trials.

**Discussion:**

These N*-of-*1 trials address an unmet medical need and will provide information on the effectiveness of CBD for severe behavioral manifestations in RGNDs, potentially generating generalizable knowledge at an individual-, disorder- and RGND population level.

**Trial registration:**

EudraCT: 2021-003250-23, registered 25 August 2022, https://www.clinicaltrialsregister.eu/ctr-search/trial/2021-003250-23/NL.

## Highlights of the study protocol

• Addressing unmet patient needs for treatment of severe behavioral manifestations of rare genetic disorders.

• Evaluation of a novel drug approved for certain rare epilepsy syndromes many of which characterized by behavioral problems.

• Innovative trial design (series of N*-of-*1 trials) in rare disorders, applying evidence-based medicine on an individual as well as group level.

• Use of novel, patient-centered and personalized outcome measures addressing (clinically) relevant items for the patient and caregivers.

## Background

Rare genetic neurodevelopmental disorders (RGNDs) affect up to 3% of the population [1]. RGNDs are often associated with intellectual disability (ID) and psychiatric comorbidity, including autism spectrum disorder (ASD), and attention-deficit hyperactivity disorder (ADHD), that may result in behavioral manifestations such as irritability, aggression, and self-injurious behavior. Often, these behavioral manifestations pose a great challenge to treating physicians and caregivers as they are refractory to standard psychological, contextual, and pharmacological interventions, and necessitate intensive individual guidance and care. Consequently, these manifestations are associated with the quality of life of not only patients, but also their families, caregivers as well as society. Therefore, there is an urgent unmet need for novel interventional treatment approaches [2].

Over the last decade, a renewed clinical interest in the use of medicinal cannabis has resulted in promising effects for several indications [3, 4], such as treatment of epilepsy [5, 6]. Recently, CBD (Epidyolex**®**) has been approved by the European Medicines Agency (EMA) to treat refractory epilepsy associated with TSC, Lennox-Gastaut syndrome and Dravet syndrome in patients aged two years and older [7]. There are also indications of efficacy of CBD for severe behavioral manifestations in ID and RGNDs [8–10], with a favorable side-effect profile compared to currently used medication, such as antipsychotics [11, 12]. Additionally, anecdotal reports of families describe a calming effect of medicinal cannabis in some children. As it will be available due to recent market approval, it is important to examine the effectiveness of CBD on behavioral manifestations in RGNDs considering the increasing interest in CBD and urge to treat behavioral problems.

The exact mechanisms of action of CBD are unknown, but previous studies suggest that CBD interacts with many signaling systems, including antagonism of GPR55, desensitization of TRPV1 channels, inhibition of adenosine reuptake, and has neuroprotective and anti-inflammatory effects [5, 6]. CBD also affects serotonin 5HT1A signal transduction, gamma-aminobutyric acid (GABA) receptor signaling, and dopamine receptor signaling, processes that are implicated in behavior. Furthermore, CBD is believed to interact with the endocannabinoid system in several ways [13], which is involved in regulating a variety of physiological and cognitive processes [14].

Due to the rarity and heterogeneity of RGNDs, interventional research is challenging. In this study, three RGNDs that are characterized by severe refractory behavioral manifestations and for which CBD has been used at patients’ own initiative are Tuberous Sclerosis Complex (TSC), Sanfilippo disease or mucopolysaccharidosis type III (MPS III) and Fragile X syndrome (FXS). These are included as a result of the specific outpatient clinics at the Amsterdam UMC, and due to availability, urgency, and heterogeneity. These patient groups reflect varying neurobiological backgrounds and phenotypes. TSC, MPS III, and FXS are all associated with ID and a severe behavioral phenotype, allowing cross-disorders comparisons. This provides more insight into treatment effects and predictors for treatment response.

TSC is a multisystem, autosomal dominant disorder affecting about 1:6.000 ([15]; Orphanet) children and adults. It is caused by a pathogenic variant in one of two genes, *TSC1* (encoding hamartin) or *TSC2* (encoding tuberin) [16, 17]. TSC-associated neuropsychiatric disorders (TAND) include epilepsy (85%), ID (50%), ASD (50%), ADHD (30–50%) and behavioral problems (50%) [18, 19]. Recently, promising results on seizures were found in a randomized, double-blind, controlled trial for CBD in TSC-related seizures in patients with drug-resistant epilepsy, with good efficacy and safety [20]. The use of pharmaceutical-grade CBD in TSC, including relevant mechanism of action, efficacy and safety data, and drug-drug interactions with other anticonvulsant medication was previously described [21], and a zebrafish model of TSC has been used to examine the influence of CBD on TSC pathology [22]. However, its effect on TSC-related behavioral and cognitive manifestations has not yet been explored sufficiently [21].

MPS III is an autosomal recessive lysosomal storage disorder caused by a deficiency of 1 of 4 enzymes involved in the degradation of the glycosaminoglycan heparan sulphate. Four types, caused by deficiencies of the different enzymes, are recognized: MPS III type A, B, C and D, with MPS IIIA the most frequent subtype [23]. As a group, MPS III comprises 47% of all MPS cases diagnosed and the combined birth prevalence is 1.89 per 100.000 live births [24]. It is characterized by progressive intellectual neurologic deterioration (PIND) [25] and severe and progressive behavioral and sleeping problems including restless, destructive, chaotic, anxious and sometimes aggressive behavior [26]. There is yet no curative treatment [23]. To date, no evidence exists for the efficacy of CBD for MPS III, although a potential treatment approach has been described that focuses on modulation of the endocannabinoid system in lysosomal storage disorders including MPS III [27].

FXS is a relatively more common genetic disorder associated with ID. It is X-linked, occurs in approximately 1:4000 males and 1:8000 females, and is caused by an alteration in the recently renamed Fragile X Messenger Ribonucleoprotein 1 (*FMR1*) gene containing a CGG-repeat with repeat length exceeding 200 CGGs [28, 29]. Other manifestations include ADHD (70%), ASD (60%), and anxiety (80%). A recent trial with transdermal CBD gel showed good efficacy on irritability in children with FXS [9]. The role of the endocannabinoid system in FXS, its dysregulation due to the absence of Fragile X Messenger Ribonucleoprotein (FMRP), and the potential role of CBD has been previously described [13, 30]. The endocannabinoid system facilitates synaptic homeostasis and plasticity through the cannabinoid receptor 1 (CB1) on presynaptic terminals, resulting in feedback inhibition of neuronal signaling, which are thought to be disrupted in FXS and may be restored by CBD acting as a negative allosteric modulator of CB1 [31]. These findings suggest that the endocannabinoid system may be involved in the neurodevelopment and behavior regulation.

### Rationale for the N-*of*-1 design

Trials in RGNDs pose specific methodological challenges due to comorbidities and rarity of conditions [32, 33]. Additionally, patients with rare disorders require individualized treatments and outcome measures due to their heterogeneity and vulnerability. It is therefore difficult to conduct traditional randomized controlled trials (RCTs) to determine effectiveness. The N*-of-*1 methodology is an alternative type of RCT, providing rigorous, and the highest level of evidence of treatment effectiveness at an individual level and is consistent with the movement towards personalized care [34, 35]. N*-of-*1 studies are randomized, controlled, multiple cross-over trials within individual patients [36, 37] and enhance treatment precision when intervention effects are heterogeneous between individuals [38, 39]. In this way, structured and evidence-based treatment decisions can be made for an individual patient. N-of-1 trials are ideal for studying treatment effects that vary among patients [40, 41]. N-of-1 trials utilize repeated measurements within individuals, considering variability among and within patients [42]. Using statistical methods like mixed-effects models accommodates individual variations through specific random effects for each patient. Analyzing repeated measures separately for each patient allows these models to address inter-individual differences, offering a robust way to assess treatment effects in heterogeneous populations. Aggregating the results of several N*-of-*1 trials in different rare, complex, and heterogeneous disorders yields treatment effect estimates [43, 44] and contributes to the generalizability to future patients with these RGNDs, but potentially also to patients with other RGNDs [45].

### Aim and objectives

We aim to conduct a series of N*-of-*1 trials to obtain scientific evidence of the effectiveness of treatment with CBD for TSC, MPS III, and FXS. The primary objective is the treatment effect of CBD compared with placebo on irritability. Secondary objectives include assessment of the effect of CBD on psychiatric and behavioral manifestations, disease-specific manifestations, parental stress, seizure frequency, and adverse effects. Personalized outcome measures will be included as well to enable us to take comorbidities into account and to focus on personalized goals. It is hypothesized that CBD has positive effects on severe behavioral manifestations, although interindividual differences in treatment effect might be expected. Baseline characteristics, such as diagnosis, accurate comorbid symptoms, and CYP enzymes enable better interpretation of results and treatment response in these heterogeneous populations with diverse neurobiological and behavioral phenotypes. Thus, a detailed description of the baseline characteristics and demographic information, as well as an extensive set of outcome measures, will provide detailed information about which manifestations may specifically be affected, and help to unravel the mechanism of action of CBD in behavioral manifestations. With that knowledge, CBD may be used as a treatment for other disorders presenting with severe behavioral manifestations. The extensive set of outcome measures will ensure that all essential clinical characteristics of the included patients will be covered. Using a strong methodology, this trial could be considered as both a confirmatory trial for irritability and exploratory for other behavioral and psychiatric outcomes. The current series of trials is part of a project which aims to create more knowledge about the suitability of N*-of-*1 trials and personalized outcome measures for rare disorders in order to facilitate care as well as regulatory decision-making [46, 47].

## Methods/design

### Protocol development and patient engagement

The choice of TSC, MPS III, and FXS was based on the severe behavioral manifestations that are an important part of the phenotype and due to our experience with these patient groups. Representatives of the Dutch TSC and FXS patient advocacy organizations, caregivers of patients with TSC and FXS, and clinical experts of all patient groups played an important role in defining knowledge and care gaps, prioritizing the treatment study, selecting outcome measures and developing the current protocol. In the protocol, we have addressed concerns related to caregiver burden and patient burden of participation and issues for recruitment and retention.

The Emma Children’s Hospital at the Amsterdam University Medical Center (UMC) is the national expertise center for MPS III. As a result, we have close contact with all Dutch families with MPS III. In addition, we have a longstanding experience in the treatment of behavioral and sleeping problems in these patients. Furthermore, we have a national clinic specialized in TAND at ‘s Heeren Loo and neuropsychiatric manifestations of FXS, collaborating closely with TSC and epilepsy expertise centers of the UMC Utrecht and expertise center of the University Medical Center of Rotterdam (ENCORE; Genetic NeuroCognitive Developmental Disorders Rotterdam Erasmus Medical Center (MC)).

### Study design and duration

We have used the Standard Protocol Items: Recommendations for Interventional Trials (SPIRIT) extension for N*-of-*1 trials (SPENT) checklist that is aligned with the CONSORT (consolidated reporting items for trials) extension for N*-of-*1 trials (CENT) for developing this N*-of-*1 protocol [39].

**Fig. 1:**
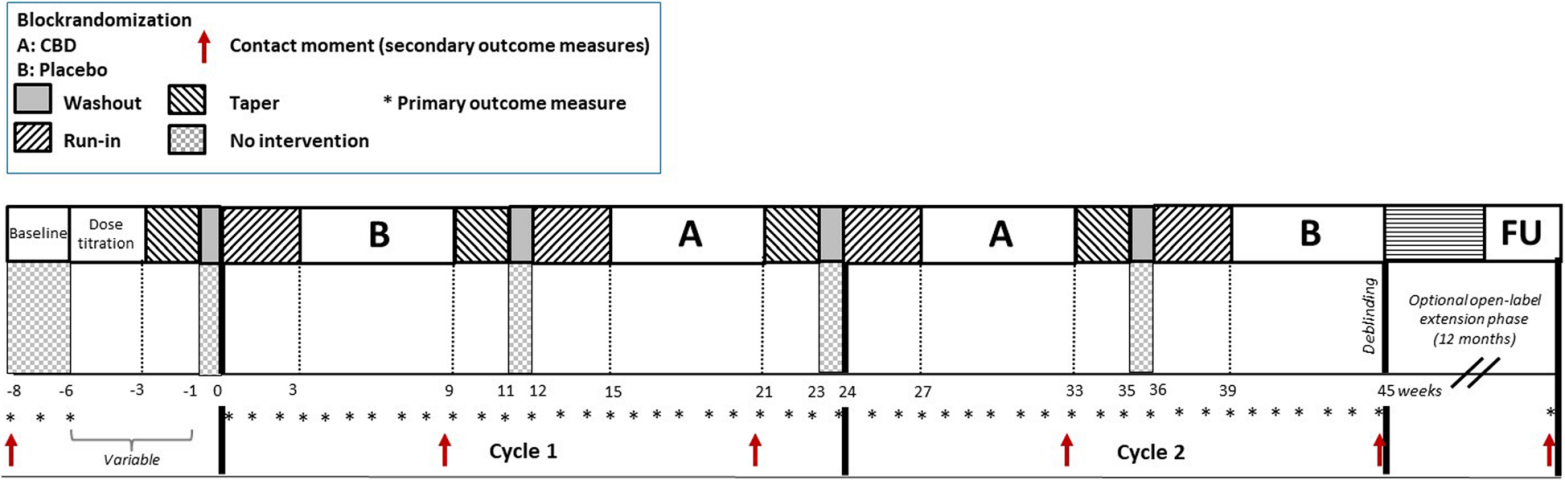
Study design of the N*-of-*trial. CBD, cannabidiol; FU, follow-up

The study will consist of a series of N*-of-*1 trials followed by an optional open-label extension phase. Each N*-of-*1 trial is block-randomized, placebo-controlled, and double-blinded with multiple crossovers in a single patient. The trial will start with a baseline period of 2 weeks without any intervention. A variable dose titration phase will follow with a taper period (2 weeks) and washout period (1 week) before starting the trial. Each N*-of-*1 trial consists of two cycles, each consisting of one period of CBD treatment (A; 6 weeks), one period of placebo treatment (B; 6 weeks), run-in periods (3 weeks), taper periods (2 weeks), and washouts following A and B (1 week) (Fig. 1). The half-life of cannabidiol in plasma is 56–61 h after twice-daily dosing for 7 days in healthy volunteers (Summary of Product Characteristics Epidyolex, https://www.ema.europa.eu/en/documents/product-information/epidyolex-epar-product-information_en.pdf). Taking this pharmacokinetic profile into account, and considering the presence of a buildup phase before the commencement of each cycle, it is expected that a 1-week washout period between cycles will adequately allow for the dissipation of any residual effects, facilitating a clear differentiation between treatment cycles.

The total duration of each trial will last around 1 year. The optional open-label extension phase will be a further 12 months.

### Study setting

Prior to the start of the trial, all clinical measures and questionnaires will be completed in the Amsterdam UMC, location AMC. The remaining questionnaires will be completed and study medication will be taken at the participants’ natural setting.

### Recruitment

Participants will be recruited through the outpatient clinics for TSC and FXS at ‘s Heeren Loo, the UMC Utrecht and the Erasmus MC, and through patient organizations and the MPS III expert center at the Amsterdam UMC.

### Study population

The study population consists of children and adults with TSC and FXS, and children with MPS III, all suffering from severe behavioral manifestations. We aim to conduct a patient-centered trial, allowing for a natural setting and flexibility, including continuation of concurrent therapies.

Inclusion criteria include:

- Minimum age of 6 years old.
- Clinically and/or genetically definite diagnosis of TSC, MPS III or FXS (modified Gomez Criteria, clinical criteria, positive genetic test, or enzyme deficiency).
- Suffering from severe behavioral manifestation with a minimum score of 4 on the Clinical Global Impression - severity (CGI-S) scale [48].
- Stable dose of all psychopharmacological medications or interventions for one month prior to screening and willingness of the participant and legal representatives to maintain the current medication regimen throughout the trial.
- Presence of a consistently available patient caregiver for proxy reports.

Exclusion criteria include:

- Any known or suspected hypersensitivity to cannabinoids or any of the excipients of the Investigational Medicinal Product (IMP), such as sesame oil.
- History of recreational or medicinal cannabis, or cannabinoid-based medications, with three months prior to screening and the patient is unwilling to abstain for the duration of the study.
- History or current evidence of significantly impaired liver function, defined as 1) Alanine aminotransferase (ALT) and/or aspartate aminotransferase (AST) > 5 x upper limit of normal (ULN); 2) ALT or AST > 3 x ULN with concomitant total bilirubin > 2.0 x ULN; or 3) ALT or AST ≥ 3 x ULN with the appearance of fatigue, nausea, vomiting, right upper quadrant pain or tenderness, fever, rash, and/or eosinophilia.
- Pregnancy or breastfeeding. Females of childbearing potential must be willing to use an effective method of contraception from the time of consent until 6 weeks after treatment discontinuation and inform the trial if pregnancy occurs.
- Glaucoma.
- History of general anesthesia in the 4 weeks prior to enrolment.
- Use of any interfering medication within 30 days prior to enrolment or planning to take interfering medication during the trial.
- Any planned major surgery within the duration of the trial.
- Expected inability to undergo blood sampling due to anxiety or resistance.
- Unwillingness or inability to swallow the study drug (or placebo).

In addition to the exclusion criteria, use of valproate should be stable 3 months prior to enrolment.

### Sample size

The sample size calculation was based on a summary measures analysis of the treatment effect [49]. The difference between the mean irritability ratings of the Aberrant Behavior Checklist (ABC) in CBD periods and placebo periods was used as a summary measure for the treatment effect in an individual participant. Heussler et al. [9] reported a standard deviation (SD) of 12 points for single ratings on the subscale. This estimate for the SD includes both within- and between-subject variance. The intraclass correlation coefficient (ICC) was assumed to be 0.75. In addition to the estimate for the within-subject variation of the outcome measure, an a priori estimate was needed for the between-subject variation of the treatment effect. Assuming an SD of 3 points for the SD of the random treatment effect, 95% of the subject-specific treatment effects roughly falls within a range of 12 points. Based on the estimate assuming two cycles with six ABC ratings within each period and assuming a two-sided significance level of 5%, a total of 6 participants will yield 80% power to detect a mean difference of 6 points between intervention and placebo periods, corresponding to a mean difference of 0.5 times the SD as reported by Heussler et al. [9]. As effects for pediatric and adult population may differ, separate power analyses were performed for the pediatric and adult cohorts. Per cohort, this amounts to 6 pediatric patients and/or 6 adult patients, with a total inclusion of 12 patients with TSC, 12 with FXS and 6 pediatric patients with MPS III. MPS III is a type of childhood dementia and most patients never reach adulthood [26]. Adult patients experience progressive dementia. This stage of the disease is not associated with severe behavioral manifestations, and therefore we will not include adult patients with MPS III.

### Blinding, treatment allocation, randomization

Participants, parents, caregivers, physicians and researchers will all be blinded during the trial. The random allocation sequence will be generated for block randomization in a 1:1 ratio and implemented by the hospital pharmacist and sequentially numbered packages. Unblinding will occur when a participant has completed the two cycles or in case of a serious adverse event (SAE) that cannot be treated without knowing which treatment the patient was receiving. Investigators involved in data analysis will remain blinded until the end of the follow-up period.

### Interventions and dosing schedule

Patients will receive a pharmaceutical formulation of highly purified CBD derived from *Cannabis sativa* L. (100 mg/mL) oral solution (Epidyolex**®** [Jazz Pharmaceuticals]) alternately with a placebo distributed by the Amsterdam UMC hospital pharmacist. CBD reduced TSC-associated seizures versus placebo with similar efficacy between the 25 and 50 mg/kg/d doses [20]. Given that the safety profile of 25 mg/kg/d was superior to 50 mg/kg/d, the lower dose range suggests a superior benefit-to-risk ratio. Standard rules for the use of CBD are in force. Participants can continue their psychopharmacological medications.

#### Dose titration phase

Prior to the N*-of-*1 trials and following the baseline period, a dose titration phase will take place, comprising escalating doses of CBD with twice daily administration from 2.5 mg/kg/day up to 25 mg/kg/day. Dose escalation steps involve an increase of 2.5 mg/kg/day. Adverse effects during the dose titration phase will be checked twice a week by a video or phone call. Also, hepatic enzyme levels will be measured at baseline and weekly from the third week of the titration phase, unless indication requires deviation. In case of adverse effects, or if the hepatic enzyme levels are ≥ 2 higher than the levels measured at baseline, the lower dose (2.5 mg/kg lower) will be taken. The final dosage will be based on the dose titration phase, with the highest dosage applied during this phase with the least adverse effects. The length of the dose titration phase will vary depending on the final dosage, followed by a taper and washout period.

#### Rescue medication

The study protocol does not specify any rescue medication to treat side effects of CBD treatment because, based on previous studies on the safety of CBD, these are usually mild at the doses used in this study. In case of SAEs, the patient will be withdrawn from the trial and appropriate treatment will be started.

### Outcome measures

The primary outcome is a change on the irritability subscale score of the Aberrant Behavior Checklist (ABC-I) during active interventional periods compared to placebo periods.

Secondary outcome measures include:

- Total ABC [50];
- CGI [48];
- Syndrome-specific outcome measures, including the TSC-specific patient-reported outcome measure (TSC-PROM) [51] and the Sanfilippo Behavior Rating Scale (SBRS) [52];
- Pediatric Quality of Life Inventory (PedsQL) [53];
- Anxiety, Depression, and Mood Scale (ADAMS) [54];
- Social Communication Questionnaire (SCQ) [55];
- Social Responsiveness Scale (SRS)-2 (when appropriate) [56];
- Short Sensory Profile (SSP-NL) [57];
- Parenting Stress Questionnaire (OBVL) [58];
- Goal Attainment Scaling (GAS) [59];
- Personal Questionnaire (PQ) [60];
- Focal and generalized seizure frequency;
- Adverse effects;
- Hepatic enzyme levels.

Baseline clinical characteristics, demographic information, medical history, results on CYP enzymes, and information regarding diagnosis will be collected and recorded in detail to enable better interpretation of results and explore factors associated with treatment response, because of the heterogeneity of the population and diverse neurobiological and behavioral phenotypes [47]. Collecting data on CYP enzymes has already been best practice and recommended to measure for refractory or difficult to treat behavioral manifestations in this population. The shortened version Vineland Adaptive Behavior Scales-III (VABS-III) [61] will be filled out by the clinicians to determine whether the SRS-2 could be filled out during the trial [62]. Optionally, functional excitation-inhibition electroencephalogram (fE/I EEG) recordings will be performed at baseline to detect changes in brain resting-state EEG for stratification of responsiveness post hoc. The fE/I EEG is optional considering the potential burden of this additional assessments, particularly in the context of the n-of-1 design. This study utilize specialized software tools, such as Matlab and the Neurophysiological Biomarker Toolbox, to preprocess and analyze fE/I EEG data. This comprehensive analysis allows us to evaluate various resting state parameters, such as power spectra, coherence, and E/I ratios [63]. fE/I EEG assessments are integral to our research, providing valuable insights into the functional connectivity and balance of neural networks, particularly relevant in individuals with neurodevelopmental disorders. Our investigation focuses on understanding the neural mechanisms underlying these conditions and their potential responses to therapeutic interventions, with a particular emphasis on E/I ratios and other EEG parameters.

#### Rationale for outcome measures

As a primary outcome measure, the ABC-I was selected. The ABC is a caregiver-completed rating scale for assessing problem behaviors in children and adults, which has robust psychometric properties in intellectually impaired and developmentally delayed populations [50, 64, 65]. The empirically derived and widely used irritability subscale consists of 15 items and comprises items reflecting temper outbursts, aggression, negative affect, and self-harm behavior. Irritability has been identified as a prominent behavioral correlate of ASD. The ABC-I has often been used as an outcome measure in treatment studies of behavioral problems in individuals with ASD and ID [66–68]. The ABC, including the irritably subscale, has been shown to be sensitive to treatment change in previous clinical trials of FXS [69, 70]. The other domains of the ABC, including lethargy/social withdrawal, stereotypic behavior, hyperactivity/noncompliance, and inappropriate speech, will serve as generalization measures to evaluate transfer effects of the intervention to a broader domain of functioning [71]. A generalization measure is an outcome measure that is related to the target behavior (irritability), for example irritability at school and at home (another setting), or interventional effects on a completely different behavior, such as less hyperactive when the target behavior is irritability.

By including an extensive set of secondary outcome measures in this study, we aim to explore the effectiveness of CBD on several behavioral and psychiatric domains as it is yet unclear which manifestations specifically respond to treatment. Moreover, we aim to explore if these outcome measures are appropriate and useful in these patient groups and in RGNDs in general. We chose outcome measures at different levels, such as disorder-specific, personalized and generalizable measures. As most of our patients will have ID, proxy-rated outcome measures applicable to children as well as adults were selected that have been psychometrically considered valid tools to measure aberrant behavior, anxiety, mood, ASD features, and parental stress in ID.

The CGI scale is a well-established rating tool applicable to all psychiatric disorders that can easily be used by the practicing clinician and provides an assessment of the clinician’s view of the patient’s global functioning prior to and after initiating a study medication [48]. The CGI has two components: the CGI-Severity (CGI-S) which rates illness severity, and the CGI-Improvement (CGI-I) which rates change from the initiation (baseline) of treatment. The CGI can track clinical progress across time and has shown to correlate well with standard, well-known research drug efficacy scales and longer, more tedious and time consuming rating instruments across a wide range of psychiatric diagnoses [72, 73]. However, the CGI is not goal-oriented and changes do not provide mechanistic insight. We will be utilizing the CGI to evaluate the overall clinical picture, encompassing various aspects of a patient’s functioning, which extend beyond behavioral domains. To address potential concerns related to inter-rater reliability, we have implemented training protocols for all investigators and clinicians involved in the study. Additionally, we will have the same rater conduct assessments for individual patients throughout the trial, which allows for a relative assessment of changes within each patient, facilitating the detection of meaningful shifts in their overall clinical presentation over time.

Additionally, available syndrome-specific outcome measures will be included for both TSC (TSC-PROM) and MPS III (SBRS). These PROMs focus on syndrome-specific targets that are of relevance to these patients and their functioning and quality of life. A recent validation study of the PROM for adults with TSC has shown that it is a reliable and valid instrument to measure the impact of the disease on functioning, which can be used in clinical and research settings to systematically gain insight into patients’ experiences [51]. It covers the physical, mental health domain, activities and participation and environmental factors, addressing the impact of specific TSC manifestations on adult patients’ health-related quality of life (QoL). The SBRS is developed to assess the behavioral phenotype in children with MPS III and its progression and results from treatment over time [52]. The SBRS is validated in 25 children with MPS IIIA, aged 2 to 18 years old [74]. As there is no specific questionnaire on QoL for FXS and children with TSC, the PedsQL will be used, which is a practical, brief, standardized, generic assessment tool to measure health-related QoL. Next to the pediatric version, an adults version exists which will be used for adults [53].

The ADAMS, considered a psychometrically sound instrument among individuals with ID [54], will be used to screen for symptoms of anxiety, depression and mood disorders.

The SCQ (originally the Autism Screening Questionnaire (ASQ)) will be used to assess the severity of ASD symptoms [75–78]. The SCQ current version will be used as it enables us to screen for ASD, to compare overall levels or severity of ASD symptoms, and to assess current ASD symptoms and change over time in both young and older children, adolescents, and adults.

The SRS-2 is a widely used measure of ASD symptoms for social behavioral problems in children and adults [56]. It may not be suitable for patients with a developmental age below four years. It will therefore be filled out when applicable, depending on the developmental age as assessed by the VABS-III during baseline [62].

The SSP is a commonly used shortened form of Dunn’s Sensory Profile caregiver questionnaire [79], containing 38 items measuring sensory features, organized into seven subscales. The total score will be used to measure sensory functioning [80].

Reducing severe behavioral manifestations may have benefits for family and caregivers. Therefore, we chose the parenting stress questionnaire (OBVL) which is applicable to children of all ages and has been validated for institutions for youth care, including mild ID as well [58].

Furthermore, GAS and the PQ enable us to focus on personalized and for participants relevant targets, also reflecting the treatment target [59, 81]. GAS is an individualized outcome measure, involving goal selection and scaling standardized to calculate the extent to which an individual’s goal is met. Patients and caretakers will select their own specific goals together with their treating physician/therapist. It is a measurement instrument that is very sensitive to change, in particular in small and heterogeneous patient populations [59]. The PQ is used as a symptom list to compare assessments of personalized goals with those measured by GAS [60]. Adding the PQ allows us to compare outcomes of standardized and personalized tools.

In case participants have epilepsy, a seizure diary will be used to evaluate change in seizure frequency.

Hepatic enzyme levels (ALT, AST, and bilirubin) will be checked to monitor adverse effects.

### Trial procedures

During the N*-of-*1 trial, the final CBD dosage as determined by the dose titration phase or placebo will be administered twice daily. During washout periods, no study medication will be taken.

#### N-*of*-1 trial: multiple crossover phase

**Fig. 2:**
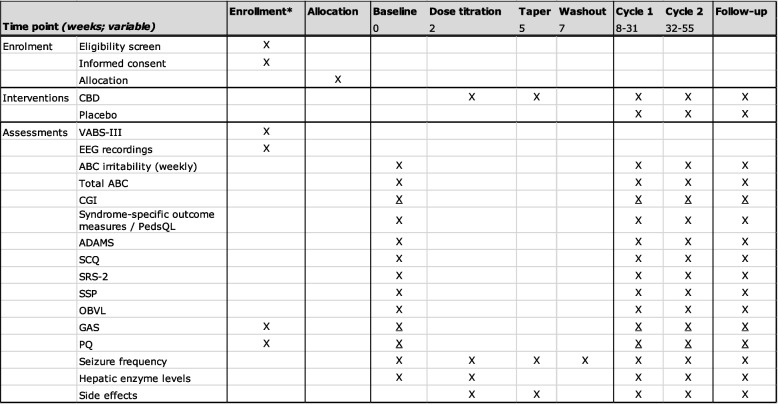
Time schedule of enrolment, interventions, and assessments. Crosses (X) indicate research steps to be conducted. Underlined crosses (X) indicate assessments via phone calls. ABC, Aberrant Behavior Checklist; ADAMS, Anxiety, Depression, and Mood Scale; CBD, cannabidiol; CGI, Clinical Global Impression; EEG, electroencephalogram; GAS, Goal Attainment Scaling; OBVL, parenting stress questionnaire; PedsQL, Pediatric Quality of Life Inventory; PQ, personal questionnaire; SCQ, Social Communication Questionnaire; SSP, Short Sensory Profile; SRS-2, Social Responsiveness Scale; VABS-III, Vineland Adaptive Behavior Scale-III

Prior to the start of the trials, the participant and substitute decision maker(s) will be seen at the clinic to discuss the procedure and sign for informed consent (Fig. 2). Personalized goals with regard to GAS will be identified together with the treating physician, psychologist, patients and primary caregivers. Seizures semiology will be discussed in detail and classified according to the classification of the International League Against Epilepsy (ILAE) [82]. Reporting of seizure frequency (by patients or caregivers) will be assessed. The shortened version VABS-III will be filled out by the clinicians to determine whether the SRS-2 could be filled out during the trial [62]. Optionally, fE/I EEG recordings will be performed at baseline.

A 2-week baseline period will follow without any intervention. The ABC, CGI, syndrome-specific outcome measures, ADAMS, SCQ, SRS-2 (if applicable), SSP-NL, and OBVL, will be filled out and seizures will be reported. These questionnaires can be completed within one hour. For children, the OBVL will be filled out by parents or primary caregivers if they have known the child for at least six months. The CGI can be completed in less than a minute by an experienced rater. A dose titration phase is followed by a taper period and washout period. During the dose titration phase, contact moments will take place (by phone) twice weekly, and hepatic enzyme levels will be measured. The individual N*-of-*1 trial consists of two cycles each containing one active treatment (A), one placebo treatment (B), run-in periods, taper periods, and washout periods. Medication will be administered at home, institution setting or day care by caregivers. The ABC irritability subscale will be filled out weekly by primary caregivers, which can be completed within a few minutes. The other outcome measures will be scored once during the baseline period, at the end of each interventional (including placebo) period, with a total of five measurements, and if participating in the optional extension phase at the follow-up measurement. Adverse effects will be assessed as well. The questionnaires can be filled out digitally (Castor EDC), phone or on paper forms. Caregivers will be asked to report all seizures in the seizure diary. To reduce burden, assessments occur by phone calls except for the clinical visits. During the experiment, it will be attempted to not switch rating caregivers.

#### Optional open-label extension phase

In consultation with the primary caregivers, patients may continue with CBD treatment during an optional one-year open-label extension phase after which a final contact moment takes place. At this follow-up measurement, questionnaires will be filled out again and personalized goals will be evaluated.

### Safety evaluation

#### Adverse events

Adverse events (AEs) will be monitored throughout the N*-of-*1 trials. All SAEs and Suspected Unexpected Serious Adverse Reactions (SUSARs) occurring during the study and either reported spontaneously or as part of the AE monitoring will be followed up by the principal investigator and will be reported separately to the Medical Ethics Committee (MEC). Unblinding will occur if there is reason to believe a SAE or SUSAR was due to the study medication and if the patient cannot be treated without knowing which treatment they were receiving. Unblinding and the reason for unblinding will be recorded. AEs, SAEs and SUSARs will be followed until they have abated or a stable situation has been reached.

#### Removal from the trial and replacement of participants

Participants will be removed from the study if informed consent is withdrawn. The investigator can decide to withdraw a participant from the N*-of-*1 trial for urgent medical reasons. Participants with hepatic enzyme level elevations sex times or greater the levels measured during baseline will be excluded, as this is a known potential for drug-induced liver injury with CBD.

In case of a drop-out, completed weeks before withdrawal will still be analyzed and a new participant will be recruited with a newly randomized sequence.

#### Premature termination of the study

The study will be terminated prematurely if more than one participant indicates a great burden of switching between placebo and CBD periods accompanied by serious safety concerns.

#### Data Safety Monitoring Board (DSMB) and monitoring

Independent qualified monitors from the Clinical Monitoring Center (CMC) will monitor the study.

Because of the small sample size and the close monitoring of patients, it was not necessary to set up a DSMB.

### Analysis

#### Data collection and management

All data will be collected and handled in accordance with applicable privacy and data protection regulations. We will collect the data according to the FAIR principles (Findable, Accessible, Interoperable, Reusable) [83]. Trial-specific documents and the Case Report Forms (CRFs) will be securely stored with restricted access limited to nominated research staff.

Assessments will be entered in the CRFs set up in Castor Electronic Data Capture (EDC). All questionnaires can be filled out digitally (Castor EDC) or by using paper forms. Automatic reminders will be sent when a questionnaire has not been completed on time. Participant burden will be limited as much as possible by using a subscale of the ABC for weekly questionnaires and having contact moments by video-conference or phone instead of a visit.

A participant identification code list will be used with unique participant identifiers not deducible to participants. Only four investigators and a methodologist and biostatistician will have access to the key and source data. Data will be stored for 25 years in a secured database and body material will be stored for 5 years.

#### Statistical methods

Individual treatment effects for participants will be quantified as summary statistics. A mixed model analysis will be applied for statistically testing the effectiveness of the intervention at group level combining data from the individual N*-of-*1 trials [84]. The mixed model will account for between-subjects heterogeneity in interventional effects by including a random treatment effect.

The mean treatment effect on the primary outcome irritability subscale of the ABC will be estimated and tested using a linear mixed model. The model will contain a fixed effect for the average treatment effect (CBD or placebo), random effects for patients, cycle within patient, and treatment (within patient). A mixed model analysis with similar model structure will be performed for the secondary study parameters. Because of the many data points per period, small amounts of missing data will not pose problems for the mixed model analysis, assuming missingness is random. The Imer package of R will be used for mixed model analyses. An analysis based on a summary measure will be performed if issues such as singularity arise. A two-sided significance level of 5% will be used.

fE/I EEG data will be processed offline using the Neurophysiological Biomarker Toolbox [85], an open-source MATLAB toolbox for the computation and integration of neurophysiological biomarkers. With this toolbox, a wide array of resting state parameters can be evaluated including power spectra and coherence and has been applied to neurological clinical studies. The data processing will be performed using MATLAB 7.12.0 software (The MathWorks Inc., Natick, MA, R2022a).

## Discussion

With the proposed series of randomized, double-blind N*-of-*1 trials with open-label extension phase, the effectiveness of CBD on severe behavioral manifestations in TSC, MPS III and FXS will be evaluated. TSC, MPS III and FXS are distinct RGNDs with unique clinical features. They share similarities in terms of their neurological involvement, ID, and behavioral challenges. However, they differ in terms of the underlying genetic variants, disease mechanisms, physical manifestations, and prevalence. It is crucial to note that each condition exhibits a wide range of symptoms and severity. Using a single study protocol for multiple disorders offers advantages such as increased efficiency, larger samples sizes, and comparative analysis opportunities, and can be considered a basket trial [86, 87]. Especially in rare, complex, and heterogeneous disorders such as these, series of N*-of-*1 trials enable determination of the treatment effects in individual patients as well as at the group level. In this way, structured and evidence-based treatment decisions can be made for an individual patient at risk for trial and error approach, and cross-over disease comparison together with medical, in-depth and mechanistic information will produce generalizable knowledge that can be applied to future patients with RGNDs.

N*-of-*1 studies are recommended in rare genetic disorders when the intervention has a predictable duration of effect and low recruitment rate is expected, like this proposed trial [47]. An explanation about the suitability of N*-of-*1 studies in RGNDs in terms of heterogeneity, personalization, design, outcome measures, and the analyses was provided in a recently published N*-of-*1 study protocol [47]. The N*-of-*1 approach to estimating population effects may come with a caveat regarding generalizability of the results. To tackle this challenge, we included three disease groups in our study. Combining the results of the N*-of-*1 trials in different patient groups potentially yields information that may be extrapolated to the RGNDs population level [45].

CBD is expensive, costing about 500 US dollars per month for rare epilepsies, although highly depending on dosages. If this study shows efficacy of CBD for severe behavioral manifestations, our major goal is to get CBD accessible to those patients who are expected to benefit, which could be facilitated by licensing and reimbursement by healthcare authorities and insurances. Before the start of the trial, we consulted ZIN (The National Health Care Institute in the Netherlands, “Zorginstituut Nederland”) and CBG (the Dutch Medicines Evaluation Board, “College ter Beoordeling van Geneesmiddelen”) on the study design, inclusion and exclusion criteria and outcome measures. Advises on outcome measures included justification for choice of the ABC-I subscale, particularly as this study aims for a broad indication of behavioral problems not associated with a specific syndrome, and defining and justifying a testing hierarchy for the endpoints with the CGI high in the testing hierarchy. An extensive list of secondary endpoints is generally accepted for an exploratory study, while limiting the number of endpoints to those relevant to support the claimed indication is recommended for a confirmatory study. Furthermore, several approaches were suggested for supporting extrapolation, such as substantiation through mechanism of action with similar effects of CBD on behavioral manifestations regardless of the neurodevelopmental disorder, subgroup analysis per disorder indicating the absence of effect modification, and inclusion of additional neurodevelopmental disorders.

The burden for the patient of the study is mostly caused by the use of blinded cross-over periods, the use of placebo, the prolonged dose titration phase, and the filling in of questionnaires by caregivers. The benefits of the study include the fact that patient-centered N*-of-*1 studies may help individuals to better self-manage their behavioral symptoms. The patients involved in the N*-of-*1 trials may draw immediate benefit from the trial as every patient is exposed to the treatment with CBD, and the N*-of-*1 design will enable an individual treatment decision in terms of evidence-based medicine. This is unlike many population-based trials where, depending on the protocol and design used, an individual may have been on a placebo for the entire trial. Moreover, data from the current series of N*-of-*1 trials will be pooled to obtain a population treatment effect estimate.

In conclusion, we consider that the N*-of-*1 trial design is excellent to study pharmacological treatments of disease manifestations in rare populations. The current study will provide crucial information about the efficacy of CBD for severe behavioral manifestations in these complex and vulnerable patient populations.

## Data Availability

Not applicable.

## Abbreviations

5HT1A: 5-Hydroxytryptamine receptor 1A
ABC: Aberrant Behavior Checklist
ABC-I: Aberrant Behavior Checklist irritability subscale
ADAMS: Anxiety, Depression, and Mood Scale
ADHD: Attention-deficit hyperactivity disorder
AE: Adverse event
ALT: Alanine aminotransferase
ASD: Autism spectrum disorder
ASQ: Autism Screening Questionnaire
AST: Aspartate aminotransferase
CB1: Cannabinoid receptor 1
CBD: Cannabidiol
CBG: College ter Beoordeling van Geneesmiddelen (Dutch Medicines Evaluation Board)
CENT: CONSORT extension for N-*of*-1 trials
CGI: Clinical Global Impression
CGI-I: Clinical Global Impression Improvement
CGI-S: Clinical Global Impression Severity
CMC: Clinical Monitoring Center
CONSORT: Consolidated reporting items for trials
CRF: Case Report Form
CYP: Cytochrome P450
DSMB: Data safety monitoring board
EDC: Electronic Data Capture
EEG: Electroencephalogram
EMA: European Medicines Agency
ENCORE: Genetic NeuroCognitive Developmental Disorders Rotterdam Erasmus Medical Center
FAIR: Findable, Accessible, Interoperable, Reusable
FMR1 gene: Fragile X Messenger Ribonucleoprotein 1 gene
FMRP: Fragile X Messenger Ribonucleoprotein
FXS: Fragile X syndrome
GABA: Gamma-aminobutyric acid
GAS: Goal Attainment Scaling
GPR55: G-protein-coupled receptor 55
ICC: Intraclass correlation coefficient
ID: Intellectual disability
ILAE: International League Against Epilepsy
IMP: Investigational Medicinal Product
MEC: Medical Ethics Committee
MPS III: Mucopolysaccharidosis type III
OBVL: Parenting Stress Questionnaire
PedsQL: Pediatric Quality of Life Inventory
PIND: Progressive intellectual neurologic deterioration
PQ: Personal Questionnaire
PROM: Patient-reported outcome measures
QoL: Quality of life
RCT: Randomized controlled trial
RGND: Rare genetic neurodevelopmental disorder
SAE: Serious adverse event
SBRS: Sanfilippo Behavior Rating Scale
SCQ: Social Communication Questionnaire
SD: Standard deviation
SPENT: SPIRIT extension for N-*of*-1 trials
SPIRIT: Standard Protocol Items: Recommendations for Interventional Trials
SRS: Social Responsiveness Scale
SSP-NL: Short Sensory Profile (Dutch)
SUSAR: Suspected Unexpected Serious Adverse Reaction
TAND: TSC-associated neuropsychiatric disorders
TRPV1: Transient receptor potential vanilloid 1
TSC: Tuberous Sclerosis Complex
ULN: Upper limit of normal
UMC: University Medical Center
VABS-III: Vineland Adaptive Behavior Scales-III
ZIN: Zorginstituut Nederland (The National Health Care Institute in the Netherlands)
